# Corrigendum: Virtual unrolling and deciphering of Herculaneum papyri by X-ray phase-contrast tomography

**DOI:** 10.1038/srep30364

**Published:** 2016-09-09

**Authors:** I. Bukreeva, A. Mittone, A. Bravin, G. Festa, M. Alessandrelli, P. Coan, V. Formoso, R. G. Agostino, M. Giocondo, F. Ciuchi, M. Fratini, L. Massimi, A. Lamarra, C. Andreani, R. Bartolino, G. Gigli, G. Ranocchia, A. Cedola

Scientific Reports 6: Article number: 2722710.1038/srep27227; published online: 06 06 2016; updated: 09 09 2016

The Acknowledgements section in this Article is incomplete.

The authors wish to thank Luigi Nicolais (CNR), Glenn Most (SNS, Pisa), Daniel Koger (Lindsey Wilson College, Kentucky, US), G. Neville Greaves (University of Cambridge, UK) for valuable discussions and revisions of the present study, the ESRF Directorate for having granted the beamtime, the Biblioteca Nazionale ‘Vittorio Emanuele III’ of Naples, for lending us the samples (in particular, the officers Sofia Maresca and Vincenzo Boni), Eugenio Amendola (CNR-IPCB), for valuable assistance in the preparation of the containers for the samples, Carlo Ionta for technical assistance in data-analysis, Luigi Verolino (University of Naples Federico II) and Gaetano Campi (CNR-IC), for preliminary discussions.

should read:

The authors wish to thank Luigi Nicolais (CNR), Glenn Most (SNS, Pisa), Daniel Koger (Lindsey Wilson College, Kentucky, US), G. Neville Greaves (University of Cambridge, UK) for valuable discussions and revisions of the present study, the ESRF Directorate for having granted the beamtime, the Biblioteca Nazionale ‘Vittorio Emanuele III’ of Naples, for lending us the samples (in particular, the officers Sofia Maresca and Vincenzo Boni), Eugenio Amendola (CNR-IPCB), for valuable assistance in the preparation of the containers for the samples, Carlo Ionta for technical assistance in data-analysis, Luigi Verolino (University of Naples Federico II) and Gaetano Campi (CNR-IC), for preliminary discussions. The European project VOXEL “Volumetric Medical X-Ray Imaging at extremely low dose” (HORIZON 2020-Fet Open; Project reference: 665207) is acknowledged for financial support. The COST action MP1203 “Advanced X-ray spatial and temporal metrology” is also acknowledged for supporting scientific missions.

