# Supplementary material for: Virtual unrolling and deciphering of Herculaneum papyri by X-ray phase-contrast tomography
Source: Sci Rep. 2016 Jun 6;6:27227. doi: 10.1038/srep27227 (PMC4893689; doi:10.1038/srep27227)
Supplement: Supplementary Information [file srep27227-s1.pdf]

Virtual unrolling and deciphering of Herculaneum papyri by X-ray phase-contrast tomography

I. Bukreeva[1,2], A. Mittone[3], A. Bravin[3], G. Festa[4,5,6], M. Alessandrelli[7], P. Coan[3,8], V. Formoso[9,10], R. G. Agostino[9,10], M. Giocondo[9], F. Ciuchi[9], M. Fratini[1], L. Massimi[1], A. Lamarra[7], C. Andreani[4,6,11], R. Bartolino[9,10,12], G. Gigli[13], G. Ranocchia[7]*, A. Cedola[1]*

Corresponding authors to: alessia.cedola@cnr.it; graziano.ranocchia@cnr.it

[1] Consiglio Nazionale delle Ricerche, Istituto di Nanotecnologia, Rome Unit, I-00195 Rome, Italy
[2] P. N. Lebedev Physical Institute, Russian Academy of Sciences, Leninskii pr., 53 Moscow, Russia
[3] European Synchrotron Radiation Facility, F-38043 Grenoble, Cedex 9, France
[4] Università degli Studi di Roma Tor Vergata, Dipartimento di Fisica, I-00133 Rome, Italy
[5] Consiglio Nazionale delle Ricerche, Istituto di Cristallografia (Bari), I-70126 Bari, Italy
[6] Museo Storico della Fisica e Centro Studi e Ricerche Enrico Fermi, I-00184 Rome, Italy
[7] Consiglio Nazionale delle Ricerche, Istituto per il Lessico Intellettuale Europeo e Storia delle Idee, I-00161 Rome, Italy
[8] Ludwig-Maximilians-Universität, Faculty of Medicine and Department of Physics, D-80799 München, Germany
[9] Consiglio Nazionale delle Ricerche, Istituto di Nanotecnologia, Cosenza Unit, I-87036 Arcavacata Di Rende (Cosenza), Italy
[10] Università della Calabria, Dipartimento di Fisica, I-87036 Arcavacata Di Rende (Cosenza), Italy
[11] Consiglio Nazionale delle Ricerche, Istituto per i Processi Chimico Fisici (Messina), I-98158, Italy
[12] Interdisciplinary Center B. Segre Accademia Nazionale dei Lincei , I-001564 Rome, Italy
[13] Consiglio Nazionale delle Ricerche, Istituto di Nanotecnologia, I-73100 Lecce, Italy

General Information

Since their discovery, numerous efforts have been made to unroll Herculaneum papyri and read the precious texts contained inside them. Initially, these attempts to unroll the scrolls failed, due to the extreme fragility of the manuscripts, and several of them were destroyed[1]. In the late 18th and 19th century the outer layers of bookrolls were usually cut off in order to expose the central windings, often less badly damaged than the exterior portions, and could be unrolled on Piaggio's machine using a sophisticated method proposed in 1756[2]. In the 1980s a chemical method based on a gelatine, acetic-acid and water solution, called the 'Oslo method', was used in further attempts to unroll the scrolls. However, this method caused most of the scrolls to break into hundreds of fragments[3]. Besides, some chemical analyses on ink were

performed[4] . Between 1999 and 2002 multispectral imaging (MSI) of all unrolled papyri was made with important results[5]. This approach showed that the writing could be clearly imaged and distinguished from the papyrus substrate with excellent spatial resolution, given the different characteristic absorption spectra of ink and papyrus and without damaging or destroying the manuscripts. This technique is now used extensively in papyrological and codicological research.

Shortly after MSI was introduced, other non-destructive techniques were applied to both papyrus scrolls and fragments, for use in reading both unrolled and rolled-up papyri. These additional techniques included, amongst other, Scanning Electron Microscopy (SEM)/EDX, Particle Induced X-ray Emission (PIXE), Computer Tomography (CT) and Nano-Computer Tomography (Nano CT), High Resolution Digital Radiography (HRDR), Thermographic Imaging (TI), X-ray Fluorescence (XRF) and Micro-CT analysis (MCT)[6]. Most recently, X-ray phase-contrast tomography (XPCT) was applied to a Herculaneum papyrus roll and a Herculaneum papyrus fragment owned by the Institute of France (Paris). This feasibility test-experiment showed the potential benefit of applying XPCT to papyri, but neither virtual unrolling nor extensive text reading was achieved[7].

Materials

The materials characterized in this study are: a) two lengthwise entire papyrus rolls (*PHerc*. 375 and *PHerc*. 495), with dimensions 7,5 cm (diameter) x 16,5 cm (height) and 7,5 cm (diameter) x 17,5 cm (height), respectively; b) a phantom sample of papyrus roll was realized using sixteen high quality sheets of bare papyrus paper originated from Sicilian papyrus plants and manufactured to reproduce the above papyrus rolls in both geometry and material. 2 cm in diameter x 10 cm in length. Sequence of numbers and Greek letters were written on the paper sheets using home-made ink. The latter was manufactured following the elemental composition used for writing on Herculanum papyri available in the literature[4]. Subsequently the paper sheets were first rolled up and then combusted - under vacuum chamber and CO_2 flow - at 350 C, a procedure which was designed to reproduce the physical condition of heat and no oxygen, the most similar to the volcanic flow of Vesuvius eruption in 79 AD.

The images of the phantom sample and of the distribution of complex components inside are shown in Figure 1S. The latter illustrates the capacity and reliability to image the contrast between the location of different fibres components and texts.
In Movie 1S one can appreciate the complex internal structure of papyrus roll *PHerc*. 495. Because of catastrophic events it is now composed of layers bent and twisted in random directions. The video shows a virtual slicing of the papyrus in the longitudinal direction, i.e. sections parallel to the long axis of the papyrus. Papyrus slices, followed during the video, represent the internal structure at different depths. Each slice has a thickness of about 50 microns and an area that covers almost all papyrus length times its diameter. Layers in the outer regions are well 'sealed', due to the thermal shock. This movie exploit the remarkable contrast and spatial resolution of XPCT experiment to its full potential.

Thanks to the XPCT technique, we discovered that *PHerc*. 495 contained a large number of grains of sand. This result revealed the history this precious scroll underwent, after being written and stored in the Library of Herculaneum. Before the thermal shock caused by the eruption of Vesuvius, it is believed that a catastrophic phenomenon, such as an earthquake, occurred. During this event *PHerc*. 495 might have been covered with sand, which penetrated inside. As the volcanic eruption took place, the thermal shock ‘sealed' the roll in its outer surface for the subsequent centuries.
Movie 2S shows a virtual slicing of the papyrus into radial direction. The sequence of images in the movie represents the sections (each about 50 microns thickness) parallel to the transversal area of the papyrus at different height. The white grains are particles of high density, i.e. the sand grains. The video shows that sand comes from the outside and it is distributed in depth.
The history of the papyri revealed to us through the XCPT analysis, is fascinating. Most exciting is the possibility to read and decipher portions of text hidden for almost two thousand years inside the bookrolls. The ‘virtual unrolling’ has revealed valuable writings by, most likely, the Epicurean philosopher and poet Philodemus of Gadara, with remarkable contrast and legibility. Both the texts and the handwriting ‘brought to light’ in the bookrolls allowed us to guess the author of both rolls and the subject matter of one of them. Movie 3S shows the virtual opening of the most intact portion of such ancient text ever found.

Supplementary Results

History and characteristics of the bookrolls

According to the *Catalogo dei papiri ercolanesi*[6], *PHerc*. 375 was partially unrolled in 1848 by Carlo Malesci through the mechanical system developed by Antonio Piaggio. The bookroll, which appears severely squeezed in height, still exhibits small traces of the thin animal membrane (‘pelle battiloro’), which was glued to the outer side of it during the unrolling. Five pieces of similar sizes for a total length of 1,725 m were obtained from this operation. These were glued upside down to paper sheets and fastened to wooden tablets. The charcoal-black color and the corrugation of the unrolled pieces seem *prima facie* compatible with those of the roll. However, the pieces’ height (max. cm 22) is quite larger (max. 5,5 cm difference) than the roll’s. This problem may be overcome only by postulating that, being the roll squeezed in height, the pieces could be stretched immediately after the unrolling. In fact, the height of the latter perfectly conforms to the standards known for unrolled Herculaneum papyri [7]. The unrolled pieces are severely layered and just a few letters are legible (Figure 2S a). Both the upper and the lower margin (blank space left by the scribe) are preserved. They contain a thoroughly unknown Greek text, which has never been transcribed nor edited.

PHerc. 495 seems to be a similar case. According to our sources [6], it would have partially been unrolled in 1820 by Humphrey Davy by applying sulfuric ether to the outermost side of the roll in order to soften it and in 1830 by Carlo Malesci through the Piaggio machine. Also in this case the roll, which appears fairly twisted and squeezed at 2/5 of its axis, still exhibits small traces of animal membrane. Ten pieces of different shapes and sizes for a total length of 1,657 m were overall unrolled and were glued to paper sheets and fastened to carton tablets. In 1987 a team led by Knut Kleve unrolled further thirteen small pieces through the chemical system known as ‘Oslo method’. Due to the unsatisfactory results of this operation the team decided to stop the unrolling. The dark-anthracite color and the corrugation of the unrolled pieces do not seem, at first sight, incompatible with those of the roll. Nevertheless, the small size of most of them does not allow a morphological comparison between them and the roll. The pieces unrolled in the XIX century exhibit some layering and in most cases the lower margin is preserved in them. For this reason and due to their limited

height (max. 13,3 cm), they must represent the lower part of a bookroll. Only some textual portions are legible (Figure 2Sb). These have been transcribed in 1820 by W. Gell and P. Elmsey, in 1853 by Vincenzo Corazza and in 1915 by Mario Arman and have been edited in 2001, together with *PHerc*. 558, by Fabio Massimo Giuliano [8]. On the contrary, the pieces unrolled in 1987, which are hardly legible, have never been transcribed nor edited. On its turn, *PHerc*. 558, consisting in 27 fragments unrolled in 1888 by Luigi Corazza and formerly taken to be the upper part of the unrolled portions of *PHerc*. 495 [9], has been proved to belong to a different bookroll on physical, paleographical and textual grounds [8]. The unrolled portions of both *PHerc*. 495 and *PHerc*. 558 contain a Greek text, which has been recognized as a *History of Socrates and his school* by Philodemus, possibly belonging to his *Arrangement of the philosophers* [8,9].

Inner structure of the bookrolls

In Figures 3Sa and 3Sc pictures of the two papyrus rolls are reported. To derive the 3D tomographic reconstruction of the internal areas inside these unopened scrolls, a software package for phase contrast tomography has been applied (see the Materials section). 3D tomographies of *PHerc*. 375 and *PHerc.* 495 are reported in Figures 3Sb and 3Sd, respectively. These show axial and longitudinal views in the 3D reconstruction, revealing the complex stress field and the layering of the material - twisted, bent and distorted - in the inner part of the scrolls. Zooms of specific areas highlight the fibre texture of the papyrus paper. The unprecedented contrast allows us to clearly distinguish and highlight portions of the bookrolls with different densities, i.e. between areas of package of sheets with different orientations and the complex individual layers. Full details of the measured distortions of the layers and changes as a function of depth inside the unopened scrolls can be seen in Movies S1. For example, one can appreciate how the outer layers in this region appear to be more closely stacked, while the interspace among carbon fibres of the papyri are drastically reduced. This was interpreted as a consequence of longer exposure of the outer portions of the rolls to thermic shock waves during the Vesuvius eruption. Indeed layers in the inner part of the rolls are better preserved, well separated and loosely rolled up. The result of 3D rendering of *PHerc*. 375 and *PHerc.* 495 are reported in Figure 4S. The full 3D tomographic images for *PHerc*. 375 and *PHerc*. 495 are plotted in Figures 4Sa-c, respectively. Images of Figures 4Sd and 4Se show very

clearly that *PHerc*. 495 exhibits a large number of impurities (yellow spots), identified as small particles of sand, embedded in its bulk (Figure 4Se). *PHerc*. 375 exhibits a small number of impurities, i.e. it looks like mostly uncontaminated. Distribution of sand particles in *PHerc*. 495 can be further seen in movie S2. Both images and movies reveal that the small particles of sand are visible in the outer layers of the scroll as well as in its bulk. This is a strong evidence that the small particles penetrated into the bookroll before these were actually exposed to thermic shock waves. These results allow the conclusion that *PHerc*. 375 was already better preserved than *PHerc*. 495 already before it was eventually exposed to the thermic shock waves of the volcanic eruption.

Textual reconstruction

The virtual unrolling of the bookrolls revealed sequences of letters, words and, for the first time, expressions and textual portions as well as a textual sign (*coronis*). Just some instances of what was found are reported in Figure 3, together with a possible textual reconstruction. Please note that these reconstructions are partly conjectural and that alternative readings are also possible. Due to the large number of letters and texts detected inside the bookrolls we were also able to reconstruct a complete Greek alphabet for each roll. These are displayed in Figure 5Sa-b.

Sequences of letters. For *PHerc.* 375 the three sequences]ενεῥ[,]ψιϲ [and]κωϲ [are displayed in Figure 3A. In the first case, we have three certain letters (*ene*) followed by a vertical stroke slightly reaching below the line, which is very likely to be a *rho*. Hence, either a single word like ἐνέργεια or ἐνεργέω or a phrase like]εν ἐρ̣[are equally possible. In the second case (*psis*), we have a substantive of the third declension in -ιϲ, -εωϲ like, e.g., ἀνάληψιϲ or πρόληψιϲ. In the third case, the sequence]κωϲ [(*kō s*) either coincides with the final portion of an adverb (-κῶϲ) or the termination of the first perfect active participle in the singular masculine nominative (-κώϲ). As far as *PHerc.* 495 is concerned, the sequences]επ[(*ep*),]ϲτ[(*st*) and]εχε[(*eche*) are clearly readable in Figure 3D. In the last case, we must either have a voice of a verb like, e.g., ἔχω or δέχομαι (or compounds) or the last component of a compound adjective of the third declension in -εχήϲ, -έϲ such as ϲυνεχήϲ or προϲεχήϲ.

Words/expressions. For *PHerc.* 375 we have selected the words τη̣ρῆι (*tē rē i*), περιε (*perie*) and π]ε̣ιϲθεῖεν̣ (*peistheien*). These are displayed in Figure 3B. The first is the

active subjunctive present of the verb τηρέω, 'watch over, preserve', or a compound of it in the 3rd singular person. The second is the passive octative aorist of πείθω, 'persuade', in the 3rd plural person. The third word is either the preposition περί or the preverb περι-, 'about' or 'around', followed by a verb or any other word beginning in ε-. As to *PHerc.* 495, the word]τείπ̣ομ[εν (*teipomen*), and the expressions]εν̣ γά[ρ (*en gar*) and]τελη βημ[α (*telē bē ma*) have been selected in Figure 3E-F. The first one is the indicative aorist, 1st plural person, of a compound of λέγω like καταλέγω, 'recount', 'enumerate' (κατείπομεν) or ἀντιλέγω, 'speak against or in reply to' (ἀντείπομεν). It is probably a *pluralis auctoris* used by the author of the book for referring to a previous section of it in which he has either told at lenght some issues or has gainsaid to an opponent of his. This usage is well attested in Philodemus. The second expression may either be a combination of particles like μ]ὲν̣ γά[ρ, 'for, on the one side', or an expression like] ἐν̣ γά[ρ or οὐδ]ὲν̣ γά[ρ or even the 3rd singular or the 1st plural person of any active verb followed by γά[ρ. The third is formed by the second component of a compound adjective in -τελής, -ές such as πολυτελής or παντηλής or, less likely, the plural noun τέλη (from τέλος, 'end' or 'purpose') and the noun βῆμα, 'tribune', in either the singular or the plural. This term means the raised place from which orators spoke in a public assembly or in a lawcourt and, significantly, is used at least twice in Philodemus' *On Rhetoric* (col. 56, 8 Sudhaus II; col. 264, 21 Sudhaus I).

Textual portions. For *PHerc.* 375 we have chosen a clearly decipherable text with an unexpectedly high contrast and legibility (Figure 3C). At line 1, we read the sequence of letters οκτο (*okto*). In an author like Philodemus (by far the most represented in the Herculanean library) this textual sequence does not seem to exist. Conversely, the sequence οκτ does exist and gives place to the words ὀκτώ, ἀποκτείνω and δέδοκται, middle perfect from δοκέω, 'think', 'seem'. All three possibilites must be excluded when adding an -ο (*omicron*) after οκτ-. However, the third one stands provided that we inflect it in the impersonal middle pluperfect ἐδέδοκτο,'it had seemed', 'it had been resolved', a quite spread form in Greek (126 occurrences in the TLG). At line 2, the succession of letters στοιοι (*stoioi*) is not possible as such in Greek. Yet, by dividing στοι from οι, we can either have a syntagm like ἕκα]στοι οἱ̣, 'each of', or, as alternatives, an adjective in the plural masculine nominative such as χρηστοί or also a superlative like πλεῖστοι followed by either a definite article (οἱ) or a relative pronoun (οἵ) agreed with the preceding adjective or also any word beginning with οι-. At line

3, parts of an *omicron* and a large *eta* are distinctly legible. As for *PHerc.* 495, among the various textual portions that we were able to identify, we have decided to present here one which may give us an idea of what is being discussed (Figure 3G). At the end of line 1, we read π̣η̣ν (*pē n*) or τ̣η̣ν (*tē n*). At line 2, we find the succession νπολ̣ι (*npoli*) that can either admit a single word like, e.g., cυνπολίτηc, 'fellow-citizen', or cυνπολιτεύω, 'live as fellow-citizens', or two different words whereof the latter must be something like πόλιc, 'city-state', πολιτικόc, 'civil' or 'political', etc. In effect, in the following line we clearly read π̣όλε̣ιc (*poleis*), 'city-states', followed by ὁ̣π[(*hop*), which may either belong to a conjunction like ὅπωc, 'as', 'how', 'that', or an adjective like ὁποῖοc, 'of what sort', or a pronoun like ὅπερ, 'what', or also an adverb like ὅπου, '(some)where' or even be divided into ὁ or ὅ followed by any word beginning with π-. In any case, the discussion seems to focus on civil or political matters. Please note that the different size and the deformation of some letters in this as well as other images is due to the optical distorsion produced by the (even extremely) irregular surface of the papyrus substrate.

Signs. We also detected inside *PHerc.* 495 a typical sign used by scribes in antiquity for signalling specific articulations of the text (Figure 3H left). It is a *coronis*, viz. a special textual marker usually indicating the end of a chapter, a book section or even a whole book. Normally, it looks like a more or less stylized sketch of a bird. So as it appears here, it roughly consists of an ellipse from which an oblique straight stroke departs on its left-bottom hand. This ellipse is superimposed on a squeezed wider oval, which appears to be open to the right. At the bottommost we find a horizontal line. Traces of other decorative strokes (possibly an upright crossed by a horizontal or an arch) are still visible at the top of the figure.

Palaeographical description

Given the large number of texts identified, it was possible to analyse the handwriting exhibited by each roll. While being aware that the irregular surface of the layers on which the letters lie may well cause optical distortions, we could make a detailed paleographical description. In this respect, we have to do with two similar, though not identical, hands. Both represent very formal round bookhands with a geometrical shaping and no modular contrast. The letters, which are ca. 2-3 mm high in an average, are accurately written and are endowed with a few decorative elements. Bilinearity is strictly followed, with the exception of *phi* and *psi*. In neither case were

ligatures detected. In particular, in *PHerc.* 375 *alpha* has the middle stroke either horizontal or ascending, *epsilon* has the middle bar outreaching the arch and either detached from or attached to it, *eta* is either epigraphical or with an ascending middle stroke joining the right vertical at the top, *kappa* has a rounded vertical stroke and is made with either very long arms or in a narrower way with the lower arm fairly suspended above the line, *my* is made in four strokes and is very angular, *zeta* and *xi* are epigraphical, *pi* is very large and has slightly divergent verticals which appear rounded at the end, *tau* is made in two strokes and is also quite large, *ypsilon* and *omega* are made in two strokes, *chi* has the descending stroke with symmetrically hooked finials. In *PHerc.* 495, *alpha* is sometimes performed more cursively, *delta* and *lambda* have the right stroke projecting above, *epsilon* has the middle bar often outreaching the arch and always attached to it, *zeta* has slightly undulated horizontals, *theta* is ogival and sometimes shows the middle bar in a higher position, *kappa* has either the lower or both arms rounded at the end, *my* is in four strokes and is very angular as in *PHerc.* 375, *xi*, *pi* and *tau* have the horizontal stroke gently rounded on the left, *pi* shows the verticals blobbed at the end and the right one often concave and suspended above the line, *ypsilon* is made in three strokes with a wide chalice and a short vertical, *omega* is either as in *PHerc*. 375 or shows slightly angular curves. On the basis of these features, it is possible to date tentatively these hands to the middle and the third quarter of I century BC, respectively. Useful parallels are, for *PHerc*. 375, those represented by *PHerc.* 1021 (belonging to G. Cavallo's Group F) and *PHerc.* 1005/862 (Group G) and, for *PHerc.* 495, that witnessed by *PHerc.* 1050 (Group I)[10]. Among Greco-Egyptian papyri, the closest parallel to *PHerc*. 375 is *POxy* XXIV 2399 (I century BC) and, for *PHerc*. 495, *PLouvre* inv. E 7733 *verso* (end of I century BC) [11]. Since Cavallo's Groups F, G and I mostly include texts by Philodemus (the others' authors being either unidentified or controversial), it is very likely that both bookrolls hand down writings by this philosopher. In particular, *PHerc*. 495, in which allusions are made to both orators and civil or political matters and is used a term (βῆμα) which is found in his *On Rhetoric*, might possibly represent a book of this large Philodemean treatise, either one partially known to us or a completely unknown one.

By comparing these hands with those of the already unrolled portions of each roll we obtain both a confirmation and a surprise. Despite their worst state of preservation, the hand of the unrolled portions of *PHerc*. 375, which has never been investigated so

far, appears compatible with that of the corresponding roll. Here as well we have to do with a highly formal, strictly bilinear and geometrical round bookhand with no modular contrast and some blobs or serifs at the end of the straight strokes. Among the single letters, only *ypsilon*, made in three strokes with high branches and a low seriffed stem, and *pi* (epigraphical) seem at first sight to be different from those of the roll. Yet, there are instances of both letters that are made in the same way as in it (*ypsilon* in two strokes with a high stem, *pi* with divergent verticals). The case of *PHerc.* 495 is more remarkable. The hand inferable from the unrolled portions bearing this number appears unquestionably different from, and hence incompatible with, that of the roll. In effect, the writing of the unrolled fragments of *PHerc.* 495, belonging to Cavallo's Group D [10], is a formal upright bookhand with a marked modular contrast and no decorative elements. *Epsilon*, *theta*, *omicron* and *sigma* are fairly narrow and angular. Not only *phi* and *psi* but also *ypsilon* and *rho* and sometimes even *tau* and *kappa* are extrabilinear. *Delta* has a convex basis, *eta* shows often a high middle bar, *my* is in either three or four strokes with a rounded paunch, the oblique stroke of *ny* joins the right vertical before the bottom of it, the *pi* has parallel and reciprocally distant verticals, the vertical stroke of *tau* encounters the horizontal on the right portion of it, *ypsilon* is made in two strokes and has a wide chalice, the ellypse of *phi* is severely triangular. These characteristics distance indisputably the hand of the unrolled portions of *PHerc.* 495 from that of the roll bearing the same number. One may wonder why this is possible. There may be two different answers to this question: either the book was written in two different hands (as is elsewhere attested in the Herculaneum library) or, more likely, was wrongly associated with these unrolled portions at some time after its partial unrolling. In the latter case, the roll would obviously contain a different text as that handed down by the unrolled portions in question.

References

1. Sider, D. *The library of the Villa dei Papiri at Herculaneum*. (Getty Publications, Los Angeles, USA, 2005).
2. Angeli, A. Lo svolgimento dei papiri carbonizzati. *Papyrologica Lupiensia* **3**, 43–84 (1994).
3. Fosse, B., Kleve, K. & Störmer, F. C. Unrolling the Herculaneum Papyri. *Cronache Ercolanesi* **14**, 9-15 (1984).
4. Störmer, F. C., Kleve, K. & Fosse, B. Ink at Herculaneum. *Cronache Ercolanesi.* **16**, 7-9 (1986)

5. Booras, S. W. & Seely, D.R. Multispectral Imaging of Herculaneum papyri. *Cronache Ercolanesi* **29**, 95-100 (1999).
6. Gigante M. *Catalogo dei papiri ercolanesi.* (Bibliopolis, Napoli, Italy, 1979).
7. Capasso M. *Manuale di Papirologia Ercolanese.* (Galatina, Congedo, Italy, 1991).
8. Giuliano F. M. *PHerc*. 495-*PHerc*. 558 (Filodemo, *Storia di Socrate e della sua scuola?*). Edizione, commento, questioni compositive e attributive. *Cronache Ercolanesi* **31**, 37-79 (2001).
9. Crönert, W. Herkulanensische Bruchstücke einer Geschichte des Sokrates und seiner Schule. *Rhein. Mus. Phil.* **57**, 285-300 (1902).
10. Cavallo G., *Libri scritture scribi a Ercolano*. (Macchiaroli, Napoli, Italy, 1983).
11. Del Corso, L. Dalla Grecia arcaica all'età romana. In. *La scrittura greca dall'antichità all'epoca della stampa: Una introduzione*. (Eds Crisci, E. & Degni, P.) 35-75 (Carocci, Roma, Italy, 2011).

FIGURE CAPTIONS

Figure1S: Modern papyrus roll. a, before and **b**, after carbonization; **c,** axial and **d**, longitudinal view of the 3D tomographic reconstruction of the internal part of the carbonized papyrus roll; **e**, text of the papyrus written (before burning) using ink mixed with Pb; **f,** virtually unrolled text of the carbonized papyrus in absorption and **g**, phase contrast.

Figure 2S: **Multispectral images of unrolled pieces**. **a**, *PHerc*. 375 ('cornice' 1) and **b**, *PHerc.* 495 ('cornice' 2). By permission of Ministero per i Beni e le Attività Culturali e del Turismo (Steven W. Booras © Biblioteca Nazionale 'Vittorio Emenuelle III', Napoli – Brigham Young University, Provo, USA). All rights reserved.

Figure 3S: **Pictures and 3D tomography of the two carbonized Herculaneum papyrus rolls**. *Top panel*: **a**, picture of *PHerc*. 375; **b**, axial (left) and longitudinal views (central) 3D reconstruction revealing the complex stress field of the inner part of the scroll, a zoom of its specific area (top right); an enhanced zoom of top right figure (bottom right) highligthing the fibre texture. *Bottom panel*: **c**, Top panel: picture of the *PHerc*. 495 scroll; **d**, axial (left) and longitudinal views (central) 3D reconstruction revealing the complex stress field of the inner part of the scroll; zoom of a specific area of previous picture, to highlight the fibre texture (right).

Figure 4S: 3D rendering images of papyrus roll. a-b, *PHerc*. 375, and **c,** papyrus roll *PHerc*. 495. **d-e,** Segmentation of *PHerc*. 495, which highlights the sand diffusion.

Figure 5S. Greek alphabet inferable from papyrus rolls. a, *PHerc*. 375 and **b,** *PHerc*. 495

MOVIE CAPTIONS

Movies S1: Virtual re-slicing of the rolls: longitudinal slices follow one another during the movie. The distortions of the layers change as a function of depth inside the rolls.

Movies S2: Little sand particles coming from outside and penetrating up at some time into the roll. Axial slices follow one another during the movie.

Movies S3: Movie of the virtual unrolling of one portion of text.

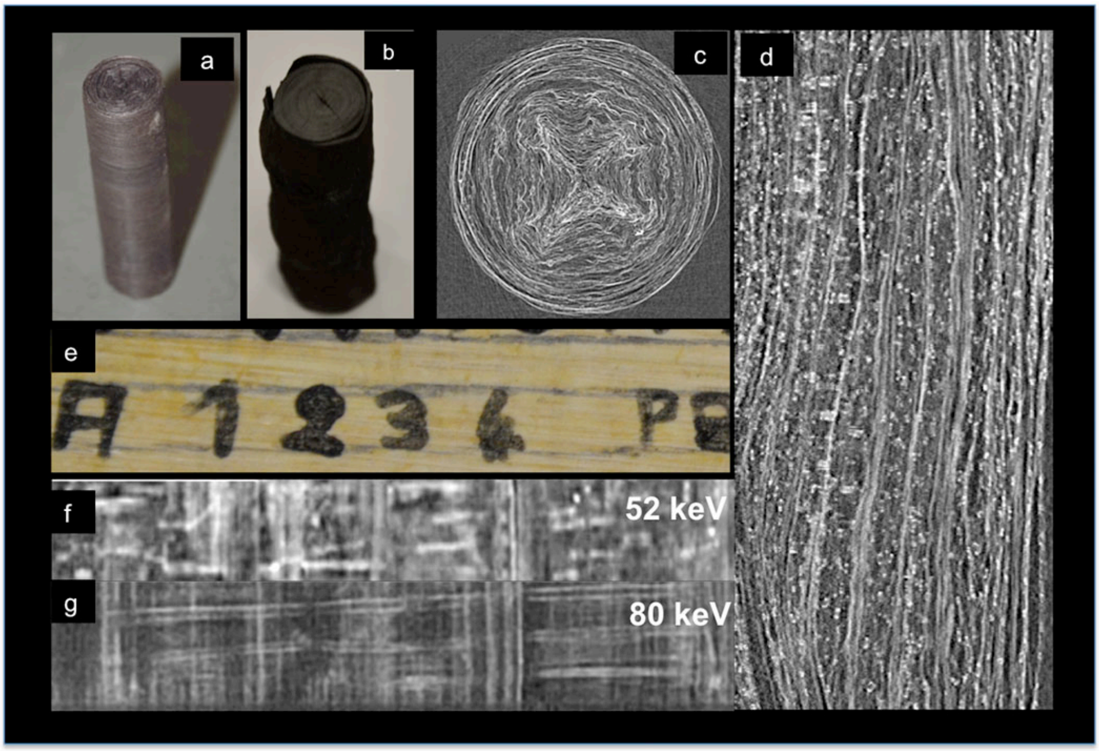

Figure1S: Modern papyrus roll. a, before and **b**, after carbonization; **c,** axial and **d**, longitudinal view of the 3D tomographic reconstruction of the internal part of the carbonized papyrus roll; **e**, text of the papyrus written using ink mixed with Pb; **f,** virtually unrolled text of the carbonized papyrus in absorption and **g**, phase contrast.

a **b**

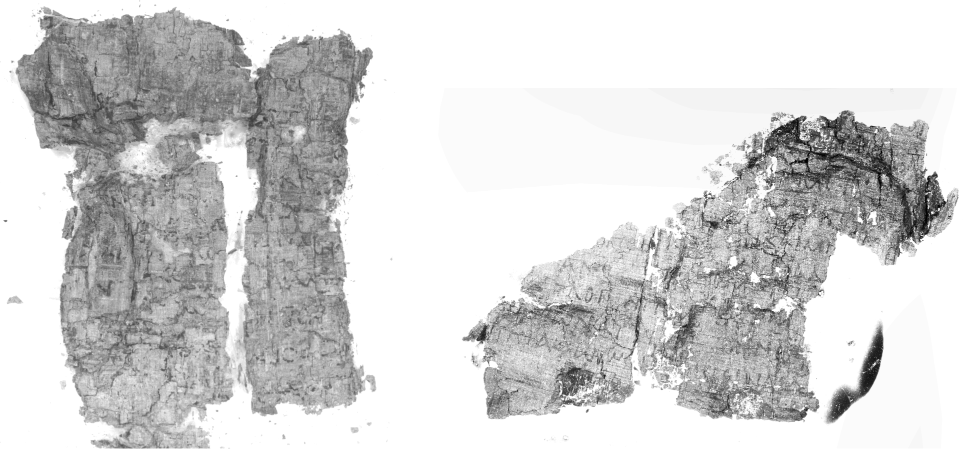

Figure 2S: Multispectral images of unrolled pieces. **a**, *PHerc*. 375 (‘cornice’ 1) and **b**, *PHerc*. 495 (‘cornice’ 2). By permission of Ministero per i Beni e le Attività Culturali e del Turismo (Steven W. Booras © Biblioteca Nazionale ‘Vittorio Emenuelle III’, Napoli – Brigham Young University, Provo, USA). All rights reserved.

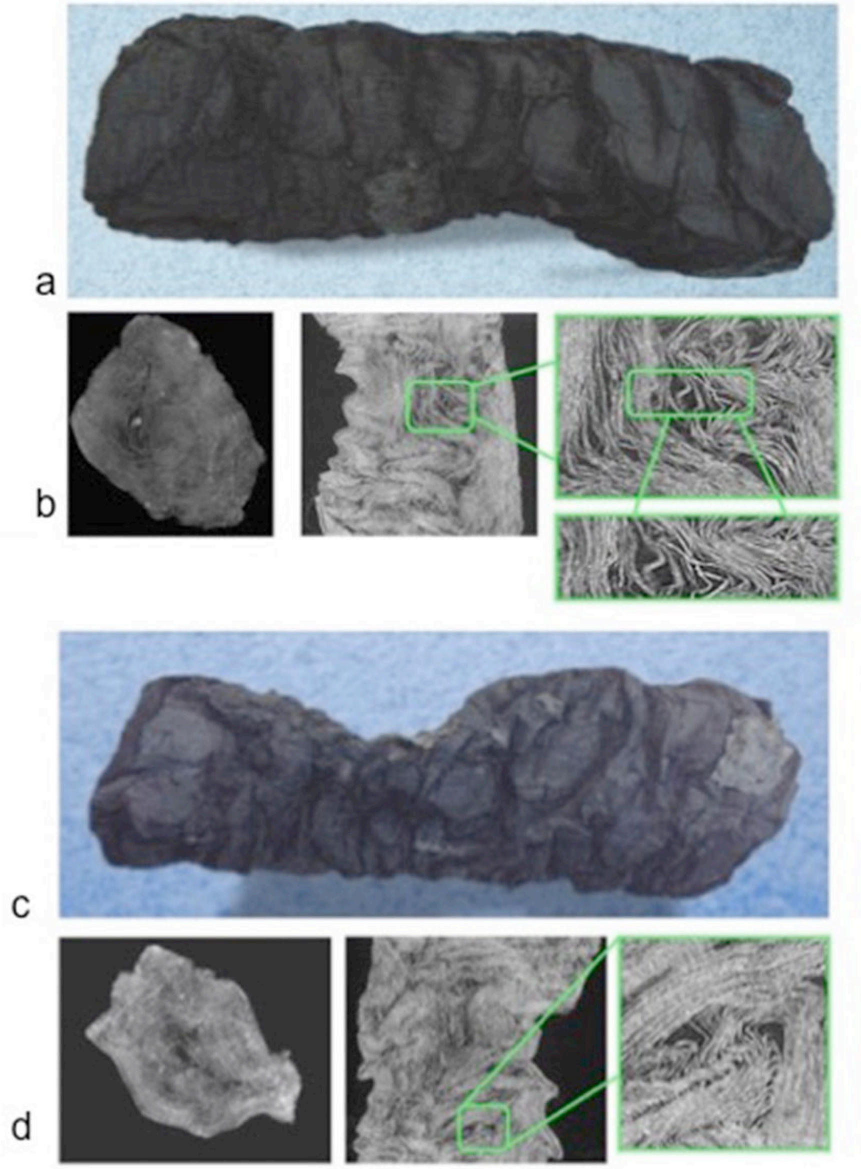

Figure 3S: **Pictures and 3D tomography of the two carbonized Herculaneum papyrus rolls**. *Top panel*: **a**, picture of *PHerc*. 375; **b**, axial (left) and longitudinal views (central) 3D reconstruction revealing the complex stress field of the inner part of the scroll, a zoom of its specific area (top right); an enhanced zoom of top right figure (bottom right) highligthing the fibre texture. *Bottom panel*: **c**, Top panel: picture of the *PHerc*. 495 scroll; **d**, axial (left) and longitudinal views (central) 3D reconstruction revealing the complex stress field of the inner part of the scroll; zoom of a specific area of previous picture, to highlight the fibre texture (right).

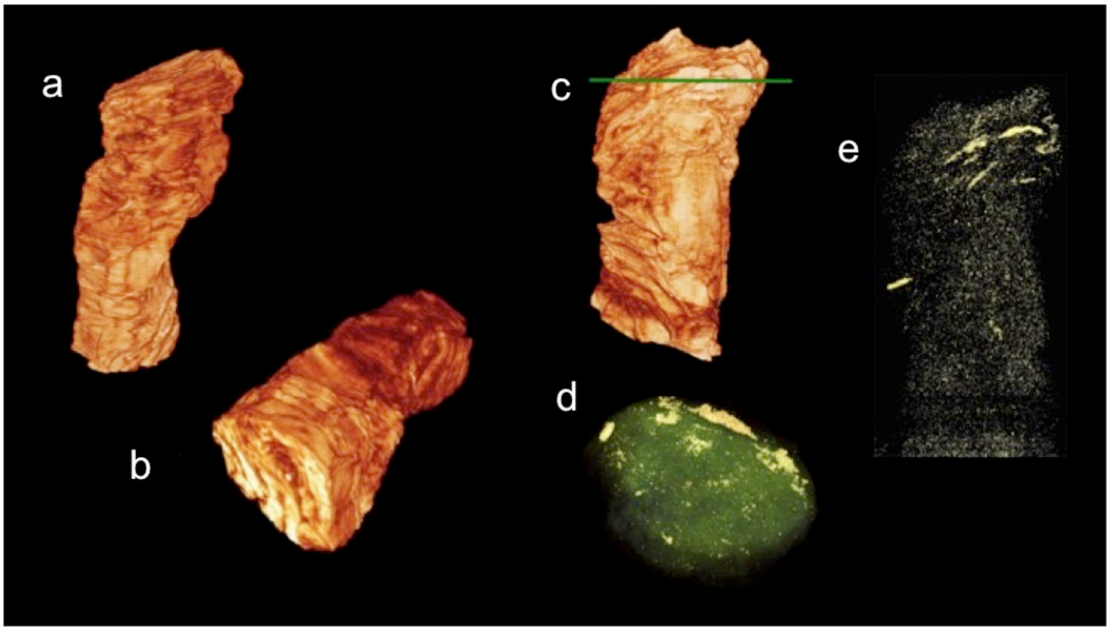

Figure 4S: 3D rendering images of papyrus roll. a-b, papyrus roll *PHerc*. 375, and **c,** papyrus roll *PHerc*. 495. **d-e,** Segmentation of *PHerc*. 495, which highlights the sand diffusion.

a

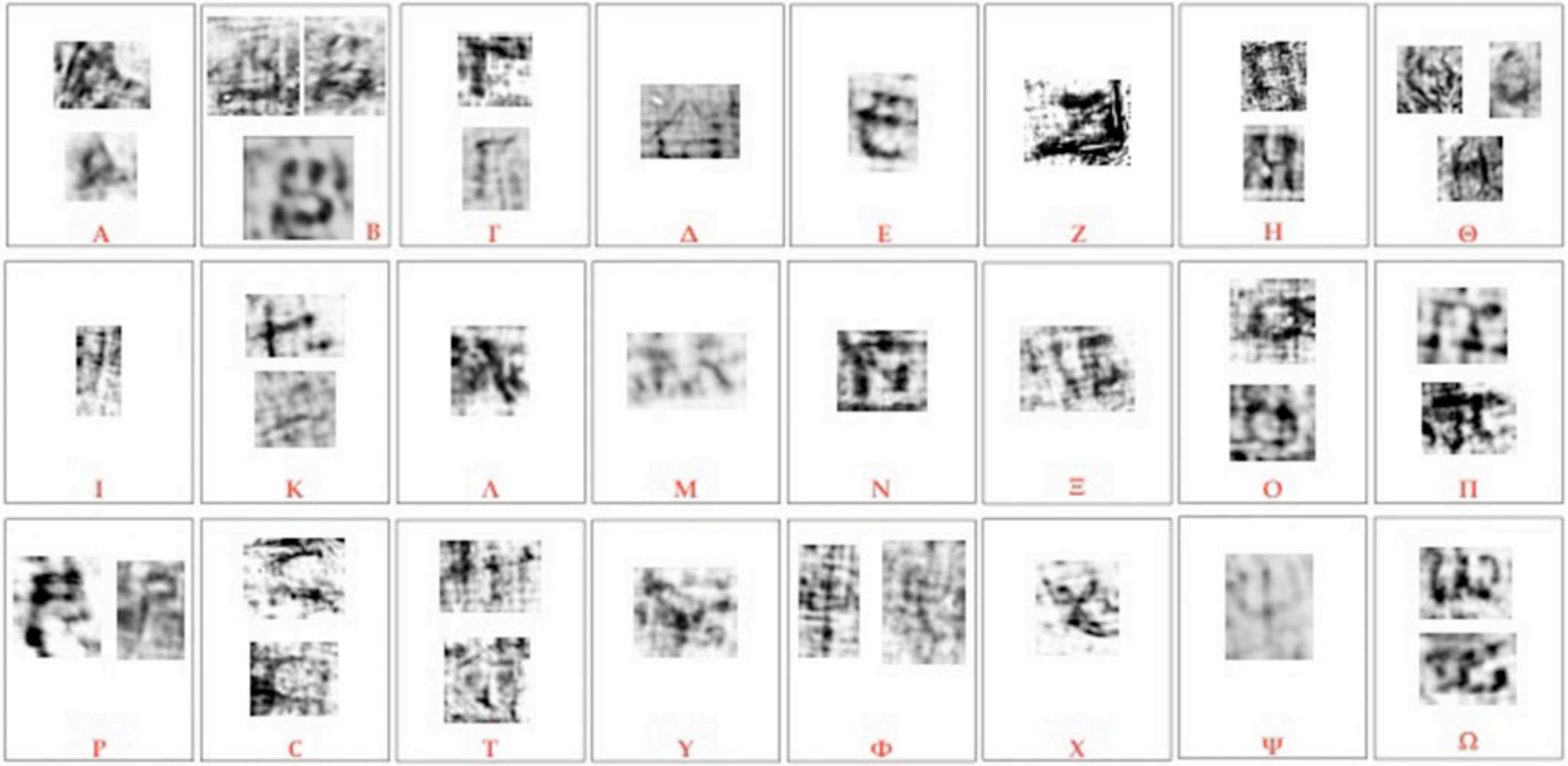

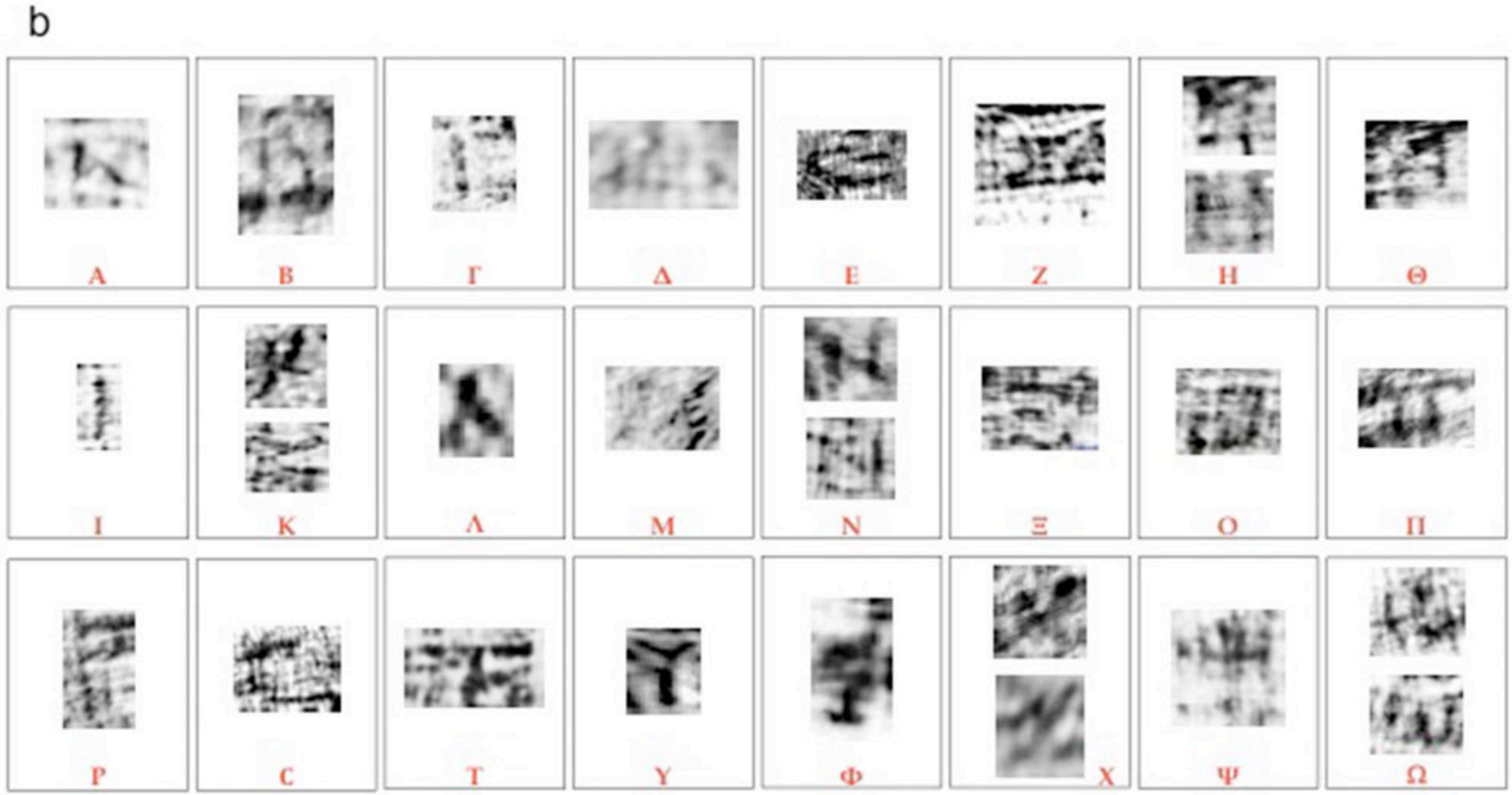

Figure 5S. Greek alphabet inferable from papyrus rolls. a, *PHerc*. 375 and **b,** *PHerc*. 495
